# Comparison of Energy Efficiency between Atmospheric Batch Pressure-Retarded Osmosis and Single-Stage Pressure-Retarded Osmosis

**DOI:** 10.3390/membranes13030354

**Published:** 2023-03-19

**Authors:** Dan Li, Zijing Mo, Qianhong She

**Affiliations:** 1 School of Civil and Environmental Engineering, Nanyang Technological University, 50 Nanyang Avenue, Singapore 639798, Singapore; dan005@e.ntu.edu.sg (D.L.);; 2 Singapore Membrane Technology Centre, Nanyang Environment and Water Research Institute, Nanyang Technological University, 1 Cleantech Loop, Clean Tech One, #06-08, Singapore 637141, Singapore; 3 Interdisciplinary Graduate Programme, Nanyang Technological University, 50 Nanyang Avenue, Singapore 639798, Singapore

**Keywords:** pressure-retarded osmosis (PRO), single-stage pressure-retarded osmosis, batch pressure-retarded osmosis, atmospheric batch pressure-retarded osmosis, osmotic energy harvesting

## Abstract

Batch pressure-retarded osmosis (PRO) with varied-pressure and multiple-cycle operation using a pressurized variable-volume tank has been proposed as a high-efficiency osmotic energy harvesting technology, but it suffers scalability constraints. In this study, a more scalable batch PRO, namely, atmospheric batch PRO (AB-PRO), was proposed, utilizing an atmospheric tank to receive and store the intermediate diluted draw solution (DS) and a pressure exchanger to recover the pressure energy from the diluted DS before being recycled into the tank. Its performance was further compared with single-stage PRO (SS-PRO) at different flow schemes via analytic models. The results show that the AB-PRO with an infinitesimal per-cycle water recovery (*r*) approaches the thermodynamic maximum energy production under ideal conditions, outperforming the SS-PRO with lower efficiencies caused by under-pressurization (UP). However, when considering inefficiencies, a ~40% efficiency reduction was observed in AB-PRO owing to UP and entropy generation as the optimal *r* is no-longer infinitesimal. Nonetheless, AB-PRO is still significantly superior to SS-PRO at low water recoveries (*R*) and maintains a stable energy efficiency at various *R*, which is conducive to meeting the fluctuating demand in practice by flexibly adjusting *R*. Further mitigating pressure losses and deficiencies of energy recovery devices can significantly improve AB-PRO performance.

## 1. Introduction

Renewable energy, as an alternative to fossil fuels in power production to reduce carbon emissions, has gained considerable attention in recent decades [1,2]. Among various types of renewable energy, osmotic energy, also called salinity-gradient energy, which originates from the mixing of two solutions with different salinities, has raised interest owing to its huge global capacity and potential accessibility from both natural and industrial sources [3,4,5,6]. The global osmotic power generated from the mixing of river water with seawater was estimated to be 1.6–2.6 TW, of which around 980 GW could be extracted as electricity, which is comparable to that generated from hydropower [7]. Despite the enormous potential, osmotic energy has not been tapped at large scales.

**Figure 1:**
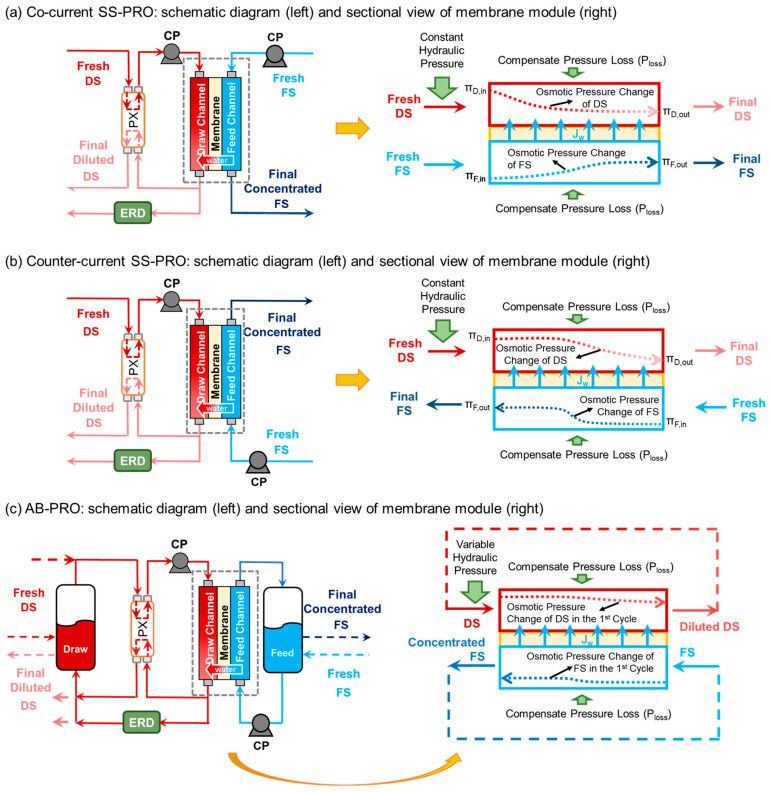
Schematic diagrams and sectional views of (**a**) co-current single-stage PRO (SS-PRO), (**b**) counter-current SS-PRO, (**c**) atmospheric batch PRO (AB-PRO). The dotted curves in the sectional views show the osmotic pressure profile along the membrane module. DS: draw solution, FS: feed solution, CP: circulation pump, PX: pressure exchanger, ERD: energy recovery device (i.e., PX or hydro-turbine), $\pi_{D,in}$ and $\pi_{D,out}$: osmotic pressure of DS influents and effluents, respectively, $\pi_{F,in}$ and $\pi_{F,out}$: osmotic pressure of FS influents and effluents, respectively.

Among various technologies for the harvesting of osmotic energy, membrane-based pressure-retarded osmosis (PRO) [8,9] and reverse electrodialysis (RED) [10,11] have the highest potential for large-scale applications. Simulated and experimental results from previous studies indicate that PRO outperforms RED in terms of higher membrane power density [12,13,14]. In PRO, a hydraulic pressure, lower than the osmotic pressure difference between a higher concentration draw solution (DS, e.g., seawater) and a lower concentration feed solution (FS, e.g., river water), is applied on the DS, which allows DS to “draw” water molecules from FS and results in a pressurized diluted DS with greater volume. In theory, net electric energy can be produced by depressurizing the increased volume of DS through a hydro-turbine. The first PRO plant with a single-stage and co-current flow configuration, as shown in Figure 1a, was designed and installed in Norway by Statkraft [15,16], in which a constant hydraulic pressure is applied throughout the operation. However, it was difficult to obtain a positive net energy production in such a PRO system in practice owing to fouling, extra energy inputs in pre-treatment stages, and inherent inefficiencies of its system design [17,18]. Figure 1a shows the sectional view of the membrane module of this co-current single-stage PRO (SS-PRO), and the dotted curves indicate the osmotic pressure profiles of DS and FS. It reveals the significant reduction of osmotic pressure difference between DS and FS at the outlet because of the simultaneous dilution of DS and concentration of FS caused by water permeation from FS to DS along the same flow direction [19]. For example, using the same volume of a 0.6 M NaCl solution (equivalent to the salinity of seawater) and a 0.01 M NaCl solution (equivalent to the salinity of river water) as the DS and FS, respectively, and assuming 90% of the water in the FS permeates to the DS during the co-current SS-PRO process, the osmotic pressure gradient shrinks from 29.2 bar at the inlet to 10.68 bar at the outlet even if salt leakage is not considered. The highest applied hydraulic pressure is constrained by the resulting low osmotic pressure difference across the membrane at the end of membrane modules. Therefore, the insufficient hydraulic pressure, also called under-pressurization (UP), especially in the first few elements, incurs irreversible energy losses.

To reduce the energy loss caused by UP, the counter-current flow mode with the opposite flow direction of the DS and FS streams is applied to PRO. As illustrated by the dotted curves in Figure 1b, the different directions of FS concentration and DS dilution make the osmotic pressure difference distributed more evenly along the membrane module, which is conducive to the application of a higher pressure, thus improving energy efficiency [20,21]. Under the same conditions discussed in the previous example, the highest hydraulic pressure that can be applied theoretically improves to 15.14 bar in the counter-current SS-PRO. More recently, a multi-stage PRO was developed to reduce energy loss by tailoring the hydraulic pressure close to the osmotic pressure difference [22,23]. A plurality of membrane modules are successively linked by the connection of their FS and/or DS inlets and the outlets of the front stage where the hydraulic pressure decreases gradually from the first to the last stage in accordance with the reducing osmotic pressure difference. However, increasing the number of stages implies additional inter-stage devices and accessories which significantly magnifies the capital cost [24,25]. To reduce energy loss and avoid increasing stages, a batch PRO (BPRO) process was proposed in which a pressurized variable-volume tank is installed on the DS side to allow the recirculation of the diluted DS to residual DS in the tank and realize the multi-cycle operation with the single-stage configuration. The osmotic pressure difference in the membrane module is more evenly distributed due to the smaller water recovery in each cycle of BPRO. The applied hydraulic pressure in BPRO varies with the osmotic pressure difference. The ideal performance of BPRO was evaluated by Li Mingheng [26], and the results demonstrated that BPRO outperforms single-stage and two- or three-stage PRO in terms of both energy production and power density, showing its potential for high-efficiency osmotic energy harvesting without additional stages and expenditures. However, in practical scenarios, scaling up the pressurized variable-volume tank remains challenging, restricting the application of BPRO [27]. Moreover, inefficiency factors, such as the pump, energy recovery device (ERD) deficiencies, and pressure drops, have yet to be considered when evaluating BPRO performance.

In this study, an alternative design of the BPRO by incorporating atmospheric tanks for storing the intermediate DS and FS and a pressure exchanger for recovering pressure energy from the DS effluent as shown in Figure 1c, namely, atmospheric batch PRO (AB-PRO), was proposed, which avoids the use of intricate pressurized variable-volume draw tank in the BPRO reported previously. Specific energy production (SEP) and energy production efficiency (EPE) of AB-PRO were calculated and compared with other PRO technologies operated in a single-stage system (i.e., co-current SS-PRO and counter-current SS-PRO) in both ideal and practical scenarios via analytical modeling.

## 2. Methods

### 2.1. Configuration and Operation

As shown in Figure 1a,b, SS-PRO is operated in an open-loop, continuous mode, where both the DS and the FS are discharged out of the system after passing through the membrane module once. The DS influent is pressurized through exchanging hydraulic pressure with the DS effluent inside a pressure exchanger (PX). Owing to the imperfect efficiency of the PX, a circulation (or booster) pump is installed to further pressurize the DS influent to the target value before it enters the membrane module. Under a constant hydraulic pressure below the osmotic pressure difference (∆*π*), water molecules permeate through the membrane from the FS to the DS, which results in a diluted DS effluent with an increased flow rate. The diluted DS effluent is split into two streams. The first stream, at the same flow rate as the DS influent, is directed to the PX to exchange pressure with the DS influent flowing to the module. The other stream at the same flow rate as the water permeating through the membrane is depressurized by an energy recover device (ERD), such as a hydro-turbine (HT) or PX, to produce electricity or pressurize a fluid in anther system [28,29,30]. The DS and the FS flowing to the membrane module can follow two different flow schemes, the co-current mode (Figure 1a) and the counter-current mode (Figure 1b).

In contrast, as shown in Figure 1c, AB-PRO is operated in a closed-loop, variable-pressure mode with two atmospheric tanks to store DS and FS. In this case, the intermediate diluted DS and the concentrated FS are recycled and mixed with the residual solutions in the DS and FS tanks, respectively, instead of being discharged out of the system. It allows AB-PRO to be operated in multiple cycles with a smaller water recovery in each cycle (i.e., per-cycle water recovery) to achieve the total water recovery requirement. Therefore, the AB-PRO process can start at a higher hydraulic pressure because only a small portion of water passing through the membrane from FS to DS in the first cycle brings about a milder decline in the osmotic pressure difference along the membrane module than that of the SS-PRO, which is also demonstrated by the osmotic pressure profiles (dotted curves) in Figure 1. As DS is diluted and FS is concentrated from cycle to cycle, the applied hydraulic pressure in AB-PRO is gradually reduced to the same level as the constant pressure applied in SS-PRO. Owing to a higher average applied pressure, AB-PRO features less energy loss due to under-pressurization compared to SS-PRO. Moreover, the AB-PRO proposed in this study adopts a PX for the energy exchange between the influents and effluents of DS, which allows the practical atmospheric tank to be used instead of the less scalable pressurized variable-volume tank proposed in the previous study. Both co-current and counter-current flow orientations can be employed in AB-PRO, but the impact of flow schemes is insignificant since the change of osmotic pressure difference in each cycle of AB-PRO is not obvious due to the small per-cycle water recovery (*r*) as illustrated by the osmotic pressure profile in Figure 1c.

### 2.2. Derivation of Energy Production

To have an insight into the practical performance of each PRO process, the impacts of inefficiencies of devices (e.g., pump, PX, and ERD) and pressure losses on both the DS and FS sides were systematically assessed. Analytic expressions of the practical maximum specific energy production (SEP) and energy production efficiency (EPE) were derived to evaluate the energy production performance of different PRO modes under ideal and practical conditions. SEP is defined as the energy generated per total volume of DS and FS, and EPE refers to the ratio of the energy extracted by PRO to the Gibbs free energy generated from the mixing of DS and FS. To simplify these analyses, it was assumed that the salt rejection of the membranes is 100%. The osmotic pressure (*π*) was assumed to be linearly proportional to the salt concentration (*c*), which can be expressed by the van’t Hoff equation [27,31]:

(1) $$\pi =vR_{g}Tc$$

where v is the number of ionic species each solute molecule dissociates into, $R_{g}$ is the ideal gas constant, and *T* is the absolute temperature, which is assumed to be 298 K in the following calculations.

#### 2.2.1. Specific Gibbs Free Energy

The Gibbs free energy of mixing ($∆G_{m}$) normalized by the total volume of DS and FS defines the thermodynamic maximum specific energy production ($SEP_{theomo}$). As illustrated in Equations (2)–(4) [19,32], the $SEP_{theore}$ is related to the concentration of FS and DS, the water recovery, and the volumetric fraction of DS and FS.

(2) $$SEP_{theomo}=\frac{∆G_{m}}{V_{F,0}+V_{D,0}}=\pi_{D,0}\left(1-\emptyset \right)\ln\left(1+\frac{\emptyset }{1-\emptyset }R\right)+\emptyset \pi_{F,0}\ln\left(1-R\right)$$

(3) $$\emptyset =\frac{V_{F,0}}{V_{F,0}+V_{D,0}}$$

(4) $$R=\frac{V_{P,T}}{V_{F,0}}$$

where $V_{D,0}$ and $V_{F,0}$ are the initial volumes of DS and FS, respectively, $\pi_{D,0}$ and $\pi_{F,0}$ are the initial osmotic pressures of DS and FS, respectively, *R* is the water recovery defined as the ratio of total water permeation volume ($V_{P,T})$ to $V_{F,0}$, which ranges from 0 to 1 (when FS is pure water), and ∅ is the volumetric fraction of FS. It should be noted that ∅ was optimized according to Equation (A1) in Appendix A in the following calculations.

#### 2.2.2. Energy Production Performance of SS-PRO

In SS-PRO, the highest constant hydraulic pressure ($P_{C}$) equals to the lowest osmotic pressure difference (∆π) along the membrane module. For a given R and ∅ in SS-PRO, the practical maximum SEP ($SEP_{SS-PRO}$) is obtained at the highest applied constant pressure. Therefore, $SEP_{SS-PRO}$ can be computed via Equation (5), and the SEP under ideal conditions ($SEP_{SS-PRO,ideal}$) can be obtained by Equation (6).

(5) $$SEP_{SS-PRO}=\eta_{ERD}R\emptyset P_{C}-\frac{\left(1-\emptyset \right)}{\eta_{P}}P_{C}+\frac{\left(1-\emptyset \right)\eta_{PX}}{\eta_{P}}P_{C}-\left[\frac{\emptyset }{\eta_{P}}P_{F,loss}+\frac{\left(1-\emptyset \right)}{\eta_{P}}P_{D,loss}\right]$$

(6) $$SEP_{SS-PRO,ideal}=R\emptyset P_{C}$$

where $\eta_{P}$, $\eta_{PX}$, and $\eta_{ERD}$ are the efficiencies of pump PX and ERD, respectively, and $P_{D,loss}$ and $P_{F,loss}$ are the pressure losses on the DS side and the FS side, respectively. Equation (5) also reveals the four energy components contributing to SEP, the specific energy production through the ERD ($\eta_{ERD}RP_{C}$), the specific energy consumption for pumping ($-\frac{\left(1-\emptyset \right)}{\eta_{P}}P_{C}$), the specific energy recovered by the PX ($\frac{\left(1-\emptyset \right)\eta_{PX}}{\eta_{P}}P_{C}$), and the specific energy loss due to pressure drop ($\frac{\emptyset }{\eta_{P}}P_{F,loss}+\frac{\left(1-\emptyset \right)}{\eta_{P}}P_{D,loss}$).

$P_{C}$ is different with various flow schemes in SS-PRO. As shown in Figure 1a, the highest pressure in the co-current SS-PRO ($P_{C,co}$) depends on the ∆π between the final diluted DS and the final concentrated FS at the end point of the membrane module (Equation (7)):

(7) $$P_{C,\mathrm{co}}=\frac{\pi_{D,0}}{1+\frac{R\emptyset }{\left(1-\emptyset \right)}}-\frac{\pi_{F,0}}{\left(1-R\right)}$$

However, the highest pressure in the counter-current SS-PRO ($P_{C,counter}$) is determined by the smaller one between the ∆π at the two ends of the membrane module (i.e., at the outlet of FS or DS).

(8) $$P_{C,counter}=\{\begin{array}{l} P_{1}=\pi_{D,0}-\frac{\pi_{F,0}}{\left(1-R\right)},\;at\;the\;outlet\;of\;FS\;and\;when\;P_{1}<P_{2}\\ P_{2}=\frac{\pi_{D,0}}{1+\frac{R\emptyset }{\left(1-\emptyset \right)}}-\pi_{F,0},\;at\;the\;outlet\;of\;DS\;and\;when\;P_{1}>P_{2} \end{array}$$

#### 2.2.3. Energy Production Performance of AB-PRO

Unlike the constant pressure applied in SS-PRO, AB-PRO features variable-pressure according to the changing ∆π during the recirculation of DS and FS. Therefore, the hydraulic pressure applied in AB-PRO is primarily related to the per-cycle water recovery (r), rather than the total target water recovery (R). In this study, r was assumed to be constant during a batch of AB-PRO. Moreover, the spatial effects in AB-PRO were ignored by assuming a spatially invariant concentration throughout the pressure vessels and tanks at a given moment. Such an assumption facilitates the derivation of the analytic expression of SEP of the AB-PRO without compromised accuracy since the length of the pressure vessel in AB-PRO can be very short and the change of ∆π can be finished in a short time [27]. The impact of flow schemes on SEP was also ignored based on this assumption. The maximum variable pressure applied in AB-PRO is a function of the time (t) as follows:

(9) $$P_{t}=\frac{\pi_{D,0}}{\left(1+\frac{r}{\theta }\right)\left[1+\frac{\emptyset }{\left(1-\emptyset \right)}\frac{t}{\tau }\right]}-\frac{\pi_{F,0}}{\left(1-r\right)\left(1-\frac{t}{\tau }\right)}$$

(10) $$r=\frac{Q_{P}}{Q_{F}}$$

(11) $$\theta =\frac{Q_{D}}{Q_{F}}$$

(12) $$\tau =\frac{V_{F,0}}{Q_{P}}$$

where $Q_{F}$ and $Q_{D}$ are the FS and DS circulation rates, respectively, $Q_{P}$ is the water permeation rate, τ is defined as the maximum retention time of the FS under the permeate flow rate of $Q_{P}$, θ is the ratio of $Q_{F}$ to $Q_{D}$ in AB-PRO, which was assumed to be the same as that in SS-PRO.

The approximation of practical maximum SEP of AB-PRO can be obtained via Equation (13). It should be noted that the pressure loss on both sides of DS and FS in AB-PRO is assumed to be 1/7 times that of SS-PRO due to the shorter module length of AB-PRO.

(13) $$SEP_{AB-PRO}=\left[\frac{\left(\eta_{PX}-1\right)\theta \emptyset }{\eta_{P}r}+\eta_{ERD}\emptyset \right]\left[\frac{\pi_{D,0}\left(1-\emptyset \right)}{\left(1+\frac{r}{\theta }\right)\emptyset }\ln\left(1+\frac{\emptyset }{1-\emptyset }R\right)+\frac{\pi_{F,0}}{\left(1-r\right)}\ln\left(1-R\right)\right]-\frac{\emptyset R}{\eta_{P}r}\left(\theta P_{D,loss}+P_{F,loss}\right)$$

By differentiating the $SEP_{AB-PRO}$ with respect to r, the optimal r can be computed by the iterative method. Under ideal conditions, the maximum SEP (Equation (A14) in Appendix B) can be obtained with r→0, which equals the specific Gibbs free energy of mixing (Equation (2)).

The SEP of different PRO processes was compared under different scenarios. The values of parameters, including $\eta_{P}$, $\eta_{PX}$, $\eta_{ERD}$, $P_{D,loss}$, and $P_{F,loss}$, are listed in Table 1.

## 3. Results and Discussions

### 3.1. Ideal Energy Production Performance

**Figure 2:**
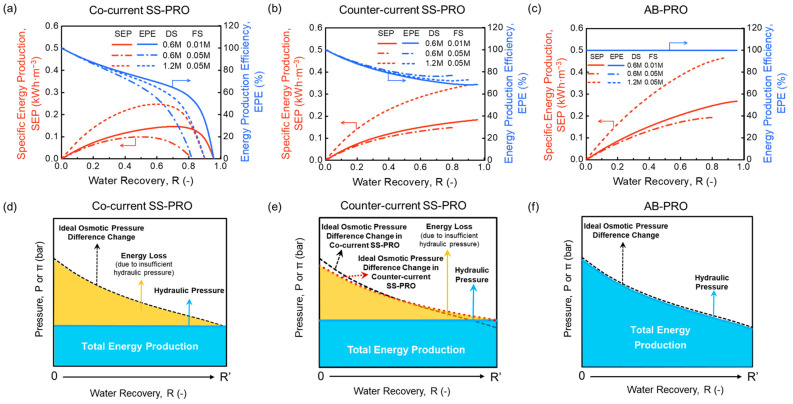
Specific energy production (SEP) and energy production efficiency (EPE) of (**a**) co-current single-stage PRO (SS-PRO), (**b**) counter-current SS-PRO, and (**c**) atmospheric batch PRO (AB-PRO), and energy components of (**d**) co-current SS-PRO, (**e**) counter-current SS-PRO, and (**f**) AB- PRO. In figure (**a**–**c**), various salt solutions with different concentrations were used for osmotic energy harvesting including a 0.6 M NaCl solution (e.g., equivalent to the salinity of seawater) and a 1.2 M NaCl solution (e.g., equivalent to the salinity of SWRO brine) as draw solution (DS) alternatives, a 0.01 M NaCl solution (e.g., equivalent to the salinity of river water) and a 0.05 M NaCl solution (e.g., equivalent to the salinity of wastewater concentrate) as feed solution (FS) alternatives. It should be noted that the x axis, water recovery, in (**a**–**c**) is different from that in (**d**–**f**). (**a**–**c**) show the SEP and EPE performance at various target total water recoveries, while (**d**–**f**) indicate the change of osmotic pressure difference and hydraulic pressure at the real-time water recovery from 0 to the target value (*R*’).

Figure 2 shows the SEP and EPE of different PRO processes with various pairs of DS and FS. The results indicate that the SEP increases with a higher salinity gradient between the DS and the FS in all three PRO processes.

As illustrated in Figure 2a,b, both co-current flow and counter-current flow SS-PRO processes show imperfect energy production efficiency (EPE) even under ideal conditions. The constant-pressure operating mode of SS-PRO results in unavoidable energy loss caused by under-pressurization (UP), which is indicated by the yellow areas in Figure 2d,e. The energy loss increases with the increase of R, leading to the decrease of EPE. However, there are still differences between the two SS-PRO processes, which have different flow schemes. For co-current SS-PRO, as R increases, the SEP first increases to a peak then decreases. According to Equation (6), without considering the inefficiencies, the SEP of SS-PRO only depends on (1) the applied pressure ($P_{C}$), (2) the water recovery (R), and (3) the volumetric fraction of FS (∅). For a specific pair of DS and FS at the optimal ∅ (Equation (A1) in Appendix A), when R increases, there is a tradeoff between the increased loss of energy owing to the drop of $P_{C}$ and the increased release of Gibbs free energy of mixing ($∆G_{m}$) due to more water permeation. When the adverse impact of UP dominates, both SEP and EPE drop significantly with the increasing R as a result of the declining $P_{C}$ owing to the rapid decrease of osmotic pressure difference (∆π) along the module in the co-current SS-PRO. When R is maximized, the osmotic pressure of the final concentrated FS is the same as that of the final diluted DS, which means that no hydraulic pressure can be applied to extract energy although $∆G_{m}$ reaches maximum. Unlike the trend of SEP, the EPE keeps decreasing as R rises. Theoretically, to obtain a high SEP and EPE simultaneously, it is necessary to operate the co-current SS-PRO at low R using a pair of DS and FS with a high salinity gradient. For example, the SEP of 0.10 kWh·m^−3^ and the EPE of 65% at R = 0.50 using a 0.6 M NaCl solution as the DS and a 0.05 M NaCl solution as the FS can be improved to 0.14 kWh·m^−3^ and 86%, respectively, by changing the DS into a 1.2 M NaCl solution and reducing the R to 0.20. For the counter-current SS-PRO, the flow scheme induces a more even distribution of ∆π along the membrane module. As illustrated by the red dotted curve in Figure 2e describing the change of ∆π in the counter-current mode, it starts from a lower value and ends at a higher value compared to the black dash curve showing the change of ∆π in the co-current mode. It can be explained by the osmotic pressure difference profile depicted by the dotted curves in Figure 1c that the opposite directions of DS and FS flows in the counter-current SS-PRO result in a lower ∆π of ($\pi_{D,in}$ − $\pi_{D,out}$) at the DS inlet than that (∆π = $\pi_{D,in}$ − $\pi_{F,in}$) in the co-current SS-PRO, and a higher ∆π of ($\pi_{D,out}$ − $\pi_{D,in}$) at the DS outlet than that (∆π = $\pi_{D,out}$ − $\pi_{F,out}$) in the co-current SS-PRO. Since the hydraulic pressure applied in SS-PRO is constrained by the lowest ∆π, the counter-current flow mode contributes to a higher appliable hydraulic pressure and more extractable osmotic energy at the same R. Equations (7) and (8) also demonstrate a higher $P_{C,counter}$ than $P_{C,co}$ from a mathematical perspective. Therefore, the counter-current SS-PRO outperforms the co-current SS-PRO on both SEP and EPE especially at high R. In addition, as a considerable concentration change is required to bring ∆π to 0 (e.g., $\pi_{D,in}$ = $\pi_{F,out}$, or $\pi_{D,out}$ = $\pi_{F,in}$) in the counter-current SS-PRO, the situation where no pressure can be applied to harvest energy will not occur in most cases. As shown in Figure 1b, the SEP of counter-current SS-PRO is monotonically increasing with R, and the EPE remains above 70% at any R.

In contrast, as shown in Figure 2c, AB-PRO can approach the thermodynamic maximum SEP and 100% of EPE under ideal conditions, which are significantly higher than those of the two SS-PRO processes. The mechanism illustrated in Figure 2f reveals that the energy loss caused by UP can be eliminated in the AB-PRO by operating with an infinitesimal per-cycle water recovery (r), where the variable hydraulic pressure applied on the DS side is as close as possible to the changing osmotic pressure difference. Using the 1.2 M NaCl solution and 0.05 M NaCl solution as DS and FS, respectively, 0.34 kWh·m^−3^ of power can be produced in AB-PRO at R = 0.5, which is equivalent to that of a 120 m-high hydropower system.

Theoretical results suggest that AB-PRO is potentially superior to SS-PRO benefiting from the reversible mixing process and variable-pressure operation. The following sections will further assess its energy production when considering practical inefficiencies.

### 3.2. Impact of Inefficiencies

Inefficiencies as listed in Table 1, including pump efficiency ($\eta_{P}$), pressure exchanger efficiency ($\eta_{PX}$), energy recovery device efficiency ($\eta_{ERD}$), and pressure loss ($P_{loss}$) on the DS and FS sides, were considered when evaluating the practical performance of different PRO processes. The pressure drops on DS and FS sides were assumed to be the same (i.e., $P_{loss}$ = $P_{F,loss}$ = $P_{D,loss}$), and the $P_{loss}$ in AB-PRO was assumed to be 1/7 of that in SS-PRO as a shorter membrane module is allowed to be utilized in AB-PRO because of the smaller water recovery in each cycle. The parameters of $\eta_{P}$ of 0.85, $\eta_{PX}$ of 0.98, $\eta_{ERD}$ of 0.90, $P_{loss}$ of 0.2 bar in AB-PRO and $P_{loss}$ of 1.4 bar in SS-PRO were set as the baseline conditions. The impact of each parameter among $\eta_{P}$, $\eta_{ERD}$, and $P_{loss}$ was investigated by the control variate method.

#### 3.2.1. Overall Impact of Inefficiencies

**Figure 3:**
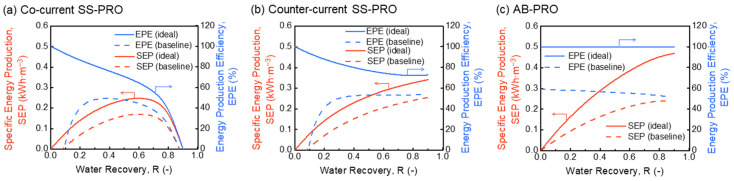
Specific energy production (SEP) and energy production efficiency (EPE) of (**a**) co-current single-stage PRO (SS-PRO), (**b**) counter-current SS-PRO, and (**c**) atmospheric batch PRO (AB-PRO) under ideal and practical baseline conditions. The feed solution (DS) is a 0.05 M NaCl solution, and the draw solution (DS) is a 1.2 M NaCl solution. Practical baseline conditions: $\eta_{P}=0.85$, $\eta_{ERD}=0.90$, $\eta_{PX}=0.98$, and $P_{loss}=0.2\;\mathrm{bar}$ in AB-PRO and 1.4 bar in SS-PRO.

As revealed by Figure 3, inefficiencies induce significant reductions in the SEP and EPE of all three PRO processes. For SS-PRO with both flow modes, the energy production cannot compensate the unavoidable energy loss caused by device inefficiencies and pressure drops in practice at low R. An R of at least 0.1 is required to gain the net energy production as indicated by Figure 3a,b. When R ranges from 0.1 to 0.25, the SEP and EPE have an insignificant difference between the co-current SS-PRO and counter-current SS-PRO, which rises with the increase of R in both SS-PRO processes. At a higher R, the EPE of the co-current SS-PRO decreases due to the rapid decrease of ∆π at the outlet of pressure vessels while that of the counter-current SS-PRO remains stable (above 50%) benefitting from the more uniform distribution of ∆π along the membrane module.

**Figure A1:**
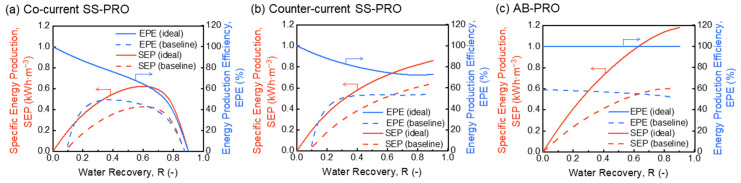
Specific energy production (SEP) normalized by initial volume of draw solution (DS) and energy production efficiency (EPE) of (**a**) co-current single-stage PRO (SS-PRO), (**b**) counter-current SS-PRO, and (**c**) atmospheric batch PRO (AB-PRO) under ideal and practical baseline conditions. The feed solution (FS) is a 0.05 M NaCl solution, and the DS is a 1.2 M NaCl solution. Practical baseline conditions: $\eta_{P}=0.85$, $\eta_{ERD}=0.90$, $\eta_{PX}=0.98$, and $P_{loss}=0.2\;\mathrm{bar}$ in AB-PRO and 1.4 bar in SS-PRO.

A significant energy loss of over 40% also occurs in AB-PRO when considering practical inefficiencies (baseline). As the r is no longer approaching 0 in practical scenarios (e.g., the optimal r is ~0.15 in the baseline case), the maximum hydraulic pressure that can be applied will be lower according to Equation (9), leading to more energy loss caused by under-pressurization (UP). Moreover, there is a larger salinity gradient between the recirculated solution and residual solution in both DS and FS tanks when r increases, hence increasing the amount of entropy generation by mixing and energy loss. Furthermore, there is a tradeoff in AB-PRO: where a higher *r* leads to an increase of energy loss caused by UP and mixing, but a reduction of energy loss by pressure drops and device inefficiencies as the total volume of solution passing through the devices and membrane module is diminished (Equations (A8) and (A9) in Appendix B). Therefore, the SEP of AB-PRO was optimized by optimizing r at each R in this study, including the data in Figure 3c and other figures showing SEP of AB-PRO in the following sections. Although AB-PRO features a similar EPE (within a 5% difference) as the counter-current SS-PRO, its SEP and EPE are significantly higher than those of both SS-PRO processes at R < 0.4. In addition, the performance of AB-PRO is relatively stable. The change of EPE with R does not exceed 10%, which facilitates the flexible adjustment of R to meet the fluctuating electricity or pressure demand in application. When R = 0.5 in the baseline case, SEP and EPE are 0.19 kWh·m^−3^ and 56%, respectively. It should be noted that the value of SEP is related to the normalization method. The total energy production is normalized by the total volume of DS and FS in this study, but the SEP can also be defined as the energy production per volume of DS or FS to show the osmotic energy harvesting capacity if FS or DS is not limited. The results normalized by the initial DS volume (Figure A1 in Appendix C) exhibit a higher SEP of 0.48 kWh·m^−3^ at R = 0.5 under the same conditions.

#### 3.2.2. Impact of Pump Efficiency

**Figure 4:**
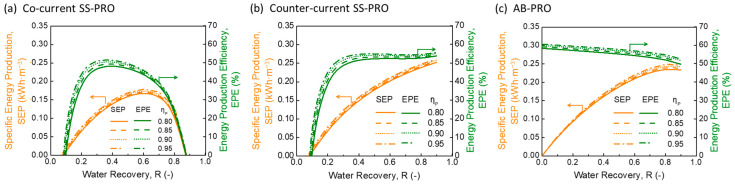
The impact of pump efficiency ($\eta_{P}$) on specific energy production (SEP) and energy production efficiency (EPE) of (**a**) co-current single-stage PRO (SS-PRO), (**b**) counter-current SS-PRO, and (**c**) atmospheric batch PRO (AB-PRO). The feed solution (DS) is a 0.05 M NaCl solution, and the draw solution (DS) is a 1.2 M NaCl solution. In addition to the different $\eta_{P}$ as shown in the figure, other conditions are fixed at the baseline level: $\eta_{PX}=0.98$, $\eta_{ERD}=0.90$, and $P_{loss}=0.2\;\mathrm{bar}$ in AB-PRO and 1.4 bar in SS-PRO.

By comparing the PRO performances at the same mode but different $\eta_{P}$, the impact of pump on SEP and EPE is not significant as demonstrated in Figure 4. In all three PRO processes, an improvement of pump efficiency from 0.80 to 0.95 only results in an increase in EPE by less than 5%. The results are mainly contributed by the configuration of a high-efficiency pressure exchanger ($\eta_{PX}$ = 0.98) which recovers most of the pressure generated by pumps and alleviates the negative effect of pump inefficiency, while the pump used in PRO is only for boosting the pressure after the PX, typically for several bars (<5 bar). The insignificant impact of $\eta_{P}$ implies more freedom in the choice of pump quality in practice. Moreover, as $\eta_{P}$ generally varies at various flow rates, the results also indicate a strong stability of the three PRO systems under different operating conditions.

#### 3.2.3. Impact of Energy Recovery Device Efficiency

**Figure 5:**
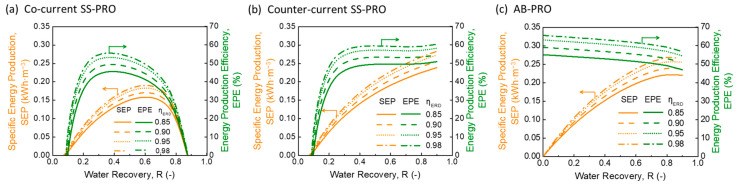
The impact of energy recovery device efficiency ($\eta_{ERD}$) on specific energy production (SEP) and energy production efficiency (EPE) of (**a**) co-current single-stage PRO (SS-PRO), (**b**) counter-current SS-PRO, and (**c**) atmospheric batch PRO (AB-PRO). The feed solution (DS) is a 0.05 M NaCl solution, and the draw solution (DS) is 1.2 M NaCl solution. In addition to the different $\eta_{ERD}$ as shown in the figure, other conditions are fixed at the baseline level: $\eta_{P}=0.85$, $\eta_{PX}=0.98$, and $P_{loss}=0.2\;\mathrm{bar}$ in AB-PRO and 1.4 bar in SS-PRO.

As shown in Figure 1, an energy recovery device (ERD), such as a hydro-turbine (HT) or a pressure exchanger (PX), is installed in PRO processes to produce electricity or transfer the pressure to another system. The impact of $\eta_{ERD}$ on the SEP and EPE is significant, as demonstrated by Figure 5. With an increase in $\eta_{ERD}$, the energy production performance is substantially improved in all three PRO processes, while the trends of performance curves for all the processes remain consistent with those in the baseline case. The maximum EPE increment in both SS-PRO and AB-PRO can approach 9% when $\eta_{ERD}$ increases from 0.85 to 0.95. By using a HT with a high efficiency of 0.95, totals of 0.19 kWh·m^−3^, 0.22 kWh·m^−3^, and 0.23 kWh·m^−3^ of electricity can be generated by the co-current SS-RO, counter-current SS-PRO, and AB-PRO, respectively, at DS = 1.2 M NaCl solution, FS = 0.05 M NaCl solution, and R=0.60. Moreover, if there is another system requiring pressure, $\eta_{ERD}$ can be further enhanced to 0.98 by replacing the HT by a PX. In this case, the EPE of ~59% can be maintained in both the AB-PRO and counter-current SS-PRO processes when R > 0.8. A higher EPE ranging from 60 to 66% can be realized in AB-PRO at R < 0.8, which is superior to that in the two SS-PRO processes, especially at low R.

#### 3.2.4. Impact of Pressure Loss

Pressure loss ($P_{loss}$) caused by the friction of fluid in the flow channel (e.g., in membrane modules) of both DS and FS induces energy loss and lessens the net energy extractable by PRO. When assuming the same optimal hydrodynamic conditions (e.g., hydraulic diameter, flow velocity, and regimes) for SS-PRO and AB-PRO, the $P_{loss}$ is proportional to the module length according to the Darcy-Weisbach equation [18,27]. In the following investigation, the $P_{loss}$ of AB-PRO was assumed to be 1/7 of that of SS-PRO (Table 1) since batch PRO can use much shorter membrane modules to achieve a similar recovery in multiple-cycle operation. Moreover, the membrane module was assumed to have high mechanical strength, hence there was no membrane deformation in the PRO processes [38].

**Figure 6:**
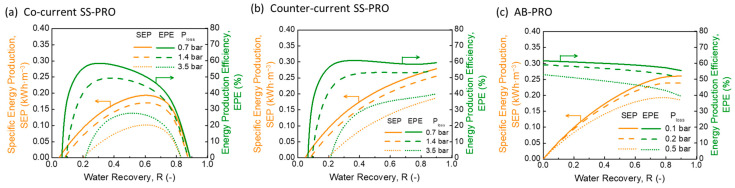
The impact of pressure loss ($P_{loss})\;$on specific energy production (SEP) and energy production efficiency (EPE) of (**a**) co-current single-stage PRO (SS-PRO), (**b**) counter-current SS-PRO, and (**c**) atmospheric batch PRO (AB-PRO). The feed solution (DS) is a 0.05 M NaCl solution, and the draw solution (DS) is a 1.2 M NaCl solution. In addition to the different $P_{loss}$ as shown in the figure, other conditions are fixed at the baseline level: $\eta_{P}=0.85$, $\eta_{PX}=0.98$, and $\eta_{ERD}=0.90$.

The results in Figure 6 indicate a significant decay of the energy harvesting performance when $P_{loss}$ increases. In SS-PRO, a higher $P_{loss}$ requires a higher R threshold to gain net energy production and leads to lower SEP and EPE at the same R. For example, if $P_{loss}$ rises from 1.4 bar to 3.5 bar, the minimal R for net energy generation increases from ~0.1 to ~0.2 in both SS-PRO processes. Meanwhile, the reduction of SEP ranges from 15% to 42% at different recoveries, and the highest EPE is only ~30% at R = 0.5 in the co-current mode and ~40% at R = 0.9 in the counter-current mode. On the contrary, the EPE can increase by 5–25% if $P_{loss}$ can be reduced to 0.7 bar in SS-PRO through optimizing hydrodynamic conditions, feed spacer geometry, and so on [38,39]. The counter-current SS-PRO always outperforms the co-current SS-PRO at various $P_{loss}$ and is capable of maintaining a relatively stable EPE of ~60% at *R* > 0.30 and $P_{loss}$ = 0.7 bar. At the $P_{loss}$ of 0.5 bar, AB-PRO presents a better performance than SS-PRO (at the $P_{loss}$ of 3.5 bar) and realizes an EPE of 40–50% benefitting from the shorter module length. However, alleviating $P_{loss}$ from 0.2 bar (baseline) to 0.1 bar only results in a 2–5% increase in EPE in AB-PRO. Although the decrease of per-cycle water recovery (r) from ~0.22 to ~0.15 because of the reduction in $P_{loss}$ brings about less energy loss caused by UP and entropy generation, it also induces more energy loss owing to inefficiencies in AB-PRO, resulting in a slight improvement of EPE. When $P_{loss}$ = 0.1 bar in AB-PRO and 0.7 bar in SS-PRO, the counter-current SS-PRO shows a small advantage compared to AB-PRO at R > 0.3, but the EPE difference between them does not exceed 3%. AB-PRO has an absolute predominance at R < 0.30.

## 4. Conclusions

The performance of atmospheric batch PRO (AB-PRO) was explored and compared with conventional single-stage PRO (SS-PRO) with different flow schemes. Variable-pressure AB-PRO with an infinitesimal per-cycle water recovery (r) can approach the thermodynamic maximum SEP and 100% of EPE under ideal conditions, while the efficiencies of two SS-PRO processes decrease with the increasing R owing to the irreversible energy loss of under-pressurization caused by the constant-pressure operation. The impact of inefficiencies, including device deficiencies and pressure losses, was also investigated for all three PRO processes. In the practical case, a significant decay of performance was observed in all three PRO processes. Although counter-current SS-PRO shows a comparable performance with AB-PRO at high R, the SEP and EPE of AB-PRO are significantly higher than that of SS-PRO at low R. AB-PRO is capable of maintaining a relatively stable and high efficiency in the entire range of R, which facilitates meeting the fluctuating energy or pressure demand in application by the flexible adjustment of R. When utilizing a 1.2 M NaCl solution as the DS and a 0.05 M NaCl solution as the FS, the SEP and EPE of AB-PRO reach 0.19 kWh·m^−3^ and 56%, respectively, in the baseline case at R = 0.5. For either AB-PRO or SS-PRO process, the advancement in pump and ERDs together with process design are critical to the further enhancement of energy production capacity. With a fixed high pressure exchanger efficiency of 0.98, pressure loss plays the most important role in the overall efficiency of PRO, followed by the ERD efficiency and pump efficiency.

## Figures and Tables

**Table 1:** Values of parameters for the energy production calculation.

Term ^a^		Ideal Case	Practical Case		Reference
			Baseline	Other Scenarios	
$\eta_{P}$		1	0.85	0.80–0.95	[31,33]
$\eta_{PX}$		1	0.98	0.98	[27,34]
$\eta_{ERD}$ ^b^		1	0.90	HT: 0.85–0.95	[35,36,37]
				PX: 0.98	
$P_{loss}$ ($P_{D,loss}=P_{F,loss}$) ^c^	SS-PRO	0 bar	1.4 bar	0.7–3.5 bar	[18,38]
	AB-PRO	0 bar	0.7 bar	0.1–0.5 bar	

^a^ $\eta_{P}$ is the pump efficiency, $\eta_{PX}$ is the pressure exchanger (PX) efficiency, $\eta_{ERD}$ is the energy recovery device (ERD) efficiency, $P_{loss}$ is the pressure loss. ^b^ the configured ERD can be either a hydro-turbine (HT) to generate electricity or a PX to exchange pressure with another system. ^c^ the pressure loss on both DS and FS sides is assumed to be the same (i.e., $P_{D,loss}=P_{F,loss}$).

## Data Availability

Not applicable.

## Abbreviations

**Abbreviation**	**Meaning**
PRO	Pressure-retarded osmosis
RED	Reverse electrodialysis
DS	Draw solution
FS	Feed solution
SS-PRO	Single-stage pressure-retarded osmosis
BPRO	Batch pressure-retarded osmosis
AB-PRO	Atmospheric batch pressure-retarded osmosis
SEP	Specific energy production
EPE	Energy production efficiency
ERD	Energy recovery device
PX	Pressure exchanger
HT	Hydro-turbine
UP	Under-pressurization

## Nomenclature

**Symbol**	**Meaning**	**Unit**
$SEP_{theomo}$	Thermodynamic maximum specific energy production	kWh·m^−3^
$SEP_{SS-PRO}$	Specific energy production of single-stage PRO	kWh·m^−3^
$SEP_{AB-PRO}$	Specific energy production of atmospheric batch PRO	kWh·m^−3^
$SEP_{SS-PRO,ideal}$	Ideal specific energy production of single-stage PRO	kWh·m^−3^
$SEP_{AB-PRO,ideal}$	Ideal specific energy production of atmospheric batch PRO	kWh·m^−3^
$∆G_{m}$	Gibbs free energy of mixing	kWh·m^−3^
$R_{g}$	Ideal gas constant	J·K^−1^·mol^−1^
v	Number of ionic species	-
T	Absolute temperature	K
∅	Volumetric fraction of feed solution	-
$\emptyset_{optimal}$	Optimal volumetric fraction of feed solution	-
R	Water recovery	-
r	Per-cycle water recovery	-
$V_{D,0}$	Initial volume of draw solution	m^3^
$V_{F,0}$	Initial volume of feed solution	m^3^
$V_{P,T}$	Total water permeation volume	m^3^
$Q_{D}$	Circulation rate of draw solution	m^3^·s^−1^
$Q_{F}$	Circulation rate of feed solution	m^3^·s^−1^
$Q_{P}$	Flow rate of permeate	m^3^/s
π	Osmotic pressure	bar
$\pi_{D,0}$	Initial osmotic pressure of draw solution	bar
$\pi_{F,0}$	Initial osmotic pressure of feed solution	bar
∆π	Osmotic pressure difference	bar
$\eta_{P}$	Pump efficiency	-
$\eta_{PX}$	Pressure exchanger efficiency	-
$\eta_{ERD}$	Energy recovery device efficiency	-
$P_{C}$	Constant hydraulic pressure	bar
$P_{C,co}$	Constant hydraulic pressure in co-current flow mode	bar
$P_{C,counter}$	Constant hydraulic pressure in counter-current flow mode	bar
$P_{t}$	Hydraulic pressure at time t	bar
$P_{loss}$	Pressure loss	bar
$P_{D,loss}$	Pressure loss in draw solution side	bar
$P_{F,loss}$	Pressure loss in feed solution side	bar
θ	Ratio of to $Q_{D}$ to $Q_{F}$	-
τ	Ratio of $V_{F,0}$ to $Q_{P}$	
$E_{pump}$	Energy consumption by pumping	kWh
$E_{PX}$	Energy recovered by pressure exchanger	kWh
$E_{loss}$	Energy consumption by compensating pressure loss	kWh
$E_{ERD}$	Energy production/recovery by energy recovery device	kWh

## Appendix A. The Optimal Volumetric Fraction

The volumetric fraction of FS was optimized to achieve the highest energy production from the mixing of FS and DS in this study. Based on Equation (1) in the main text, the optimal ∅ can be obtained by the following equation:

(A1) $$\emptyset_{optimal}=\frac{e^{\left[\frac{\pi_{F,0}\ln\left(\pi_{F,0}\right)-\pi_{D,0}\ln\left(\pi_{D,0}\right)}{\left(\pi_{F,0}-\pi_{D,0}\right)}-1\right]}-\pi_{D,0}}{\left(\pi_{F,0}-\pi_{D,0}\right)}$$

## Appendix B. Derivations of Specific Energy Production

### Appendix B.1. SS-PRO

SS-PRO is a continuous, constant-pressure process; hence the derivation of energy production is straightforward. The energy components in SS-PRO include:

(1) Energy consumption by pumping ($E_{pump}$)
  (A2) $$E_{pump}=\frac{1}{\eta_{P}}P_{C}V_{D,0}$$
(2) Energy recovered by the pressure exchanger ($E_{PX}$)
  (A3) $$E_{PX}=\frac{\eta_{PX}}{\eta_{P}}P_{C}V_{D,0}$$
(3) Energy consumption by compensating pressure loss ($E_{loss}$)
  (A4) $$E_{loss}=\frac{1}{\eta_{P}}P_{F,loss}V_{F,0}+\frac{1}{\eta_{P}}P_{D,loss}V_{D,0}$$
(4) Energy production by an energy recovery device ($E_{ERD}$)
  (A5) $$E_{ERD}=\eta_{ERD}P_{C}V_{P}$$

The SEP is the net energy production normalized by the total volume of DS and FS.

(A6)
$$SEP=\frac{-E_{pump}+E_{PX}-E_{loss}+E_{ERD}}{V_{F,0}+V_{D,0}}$$

Equation (A6) can be simplified to Equation (5) in the main text.

In co-current flow SS-PRO, $P_{c}$ is the osmotic pressure difference between the final diluted DS ($\pi_{D,f}$) and the final concentration FS ($\pi_{F,f}$) at the end point of the membrane module with $P_{C,co}=(\pi_{D,f}-\pi_{F,f})$, $\pi_{D,f}=\frac{\pi_{D,0}}{1+\frac{R\emptyset }{\left(1-\emptyset \right)}}$ and $\pi_{F,f}$ = $\frac{\pi_{F,0}}{\left(1-R\right)}$. In counter-current flow SS-PRO, $P_{C}$ is lower between the osmotic pressure difference at the FS outlet ($P_{1}=\pi_{D,0}-\pi_{F,f}=\pi_{D,0}-\frac{\pi_{F,0}}{\left(1-R\right)}$) and at the DS outlet ($P_{2}=\pi_{D,f}-\pi_{F,0}=\frac{\pi_{D,0}}{1+\frac{R\emptyset }{\left(1-\emptyset \right)}}-\pi_{F,0}$). In the ideal case, $\eta_{P}$ = $\eta_{PX}$ = $\eta_{ERD}$ = 1, $P_{F,loss}$ = $P_{D,loss}$ = 0 bar.

### Appendix B.2. AB-PRO

AB-PRO is a continuous but variable-pressure ($P_{t}$) process, hence the derivation of its energy production is based on the integration with respect to time (t). The duration time ($t_{B}$) of a batch of AB-PRO can be obtained by Equation (A7).

(A7) $$t_{B}=\frac{RV_{F,0}}{rQ_{F}}$$

It was assumed that the total water permeation rate ($Q_{P}$) is constant during the AB-PRO process. Thus, regarding a constant recirculation flow rate of FS ($Q_{F}$), r is also constant.

The volume of solution passing through the devices and membrane module on the FS side ($V_{pass,F}$) and DS side ($V_{pass,D}$) in $t_{B}$ can be obtained by Equations (A8) and (A9), respectively.

(A8)
$$V_{pass,F}=Q_{F}t_{B}=\frac{RV_{F,0}}{r}$$

(A9)
$$V_{pass,D}=Q_{D}t_{B}=\frac{\theta RV_{F,0}}{r}$$

The energy components in AB-PRO include:

(1) Energy consumption by pumping ($E_{pump}$)
  (A10) $$E_{pump}=\frac{1}{\eta_{P}}\int_{0}^{t_{B}}Q_{D}P_{t}dt$$
(2) Energy recovered by pressure exchanger ($E_{PX}$)
  (A11) $$E_{PX}=\frac{\eta_{PX}}{\eta_{P}}\int_{0}^{t_{B}}Q_{D}P_{t}dt$$
(3) Energy consumption by compensating pressure loss ($E_{loss}$)
  (A12) $$E_{loss}=\frac{1}{\eta_{P}}P_{D,loss}Q_{D}t_{B}+\frac{1}{\eta_{P}}P_{F,loss}Q_{F}t_{B}$$
(4) Energy production by energy recovery device ($E_{ERD}$)
  (A13) $$E_{ERD}=\eta_{ERD}\int_{0}^{t_{B}}Q_{P}P_{t}dt$$

The SEP of AB-PRO can also be obtained by Equation (A6) combining with Equations (9)–(12) and further simplified to Equation (13) in the main text.

In the ideal case, not only $\eta_{P}$ = $\eta_{PX}$ = $\eta_{ERD}$ = 1 and $P_{F,loss}$ = $P_{D,loss}$ = 0 bar, but also r→0. The highest SEP of AB-PRO can be approached by the following equation, which is the same as the thermodynamic maximum SEP (Equation (2) in the main text).

(A14)
$$SEP_{AB-PRO,ideal}=\pi_{D,0}\left(1-\emptyset \right)\ln\left(1+\frac{\emptyset }{1-\emptyset }R\right)+\emptyset \pi_{F,0}\ln\left(1-R\right)$$
